# Primary percutaneous coronary intervention in CAD patients: A comparison of major adverse cardiovascular events of second- and third-generation drug-eluting stents

**DOI:** 10.3389/fphar.2022.900798

**Published:** 2022-11-16

**Authors:** Salma Bibi, Amjad Khan, Amer Hayat Khan, Muhammad Niaz Khan, Saima Mushtaq, Sheikh Abdur Rashid

**Affiliations:** ^1^ Department of Pharmacy, Quaid-i-Azam University, Islamabad, Pakistan; ^2^ Discipline of Clinical Pharmacy, School of Pharmaceutical Sciences, Universiti Sains Malaysia, George Town, Penang, Malaysia; ^3^ Department of Interventional Cardiology, Hayatabad Medical Complex, Peshawar, Pakistan; ^4^ Department of Healthcare Biotechnology, Atta-ur-Rahman School of Applied Biosciences, National University of Sciences and Technology, Islamabad, Pakistan; ^5^ Gomal Center of Pharmaceutical Sciences, Faculty of Pharmacy, Gomal University, D.I.Khan, Pakistan

**Keywords:** coronary artery disease, primary percutaneous coronary intervention, major adverse cardiovascular events, XIENCE, BioMatrix

## Abstract

**Background:** Biodegradable polymer (BP) drug-eluting stents (DES) have been introduced as a novel solution to the problems of durable polymer (DP) stents. In Pakistan, very few studies are available for the treatment intervention in post-primary percutaneous coronary intervention (PPCI) patients. Our study will compare the major adverse cardiovascular events (MACEs) and their predictors in patients with coronary artery disease (CAD) undergoing PPCI with second- or third-generation DES.

**Methodology:** An observational, retrospective, cohort study was carried out on CAD patients undergoing PPCI with either second- (DP-XIENCE Prime/XIENCE Xpedition) or third-generation (BP-BioMatrix NeoFlex/BioMatrix Alpha) DES. MACEs were assessed after 1 year of PPCI procedure in 341 patients and screened as per inclusion/exclusion criteria (167 in the second-generation group and 174 in the third-generation group).

**Results:** The number of male patients (86.2%) was more than female patients in our study population. MACEs were reported in 4.19% patients after 1 year duration, and the percentage of MACEs was more in the second-generation DES group (4.77%) than in the third-generation group (3.44%); however, statistical analysis has not found any significant difference (*p* = 0.534). The rate of myocardial infarction (1.19% vs. 0.57%) and stent thrombosis (1.8% vs. 1.15%) was more in the second-generation DES group. However, restenosis (1.19% vs. 1.15%) and cardiac death (0.59% vs. 0.57%) were almost same in both groups. A significant association was found between MACEs and diabetes mellitus (*p* = 0.025), hypertension (*p* = 0.035), smoking (*p* = 0.008), and a family history of CAD (*p* = 0.018).

**Conclusion:** BP-BioMatrix and DP-XIENCE DES have comparable clinical outcomes. Findings of the current study will assist the policy makers and healthcare providers in the rationalization of scarce resources and evidence-based patient care. However, longer follow-up studies are required for convincing results.

## Highlights

• Biodegradable polymer stents are a novel solution to the problems of DP-DES and leave a polymer-free stent after releasing anti-proliferative drugs.
• To improve longevity and health after PPCI, detection of MACEs and their risk factors is very crucial.
• BP-DES have comparable/superior outcomes as compared to DP stents.
• Smoking, family history of CAD, and DM are significant predictors of MACEs.

## Background

Coronary artery disease (CAD) develops when the arteries of the heart are not able to supply enough oxygen-rich blood to the heart (National Heart, L AND Blood Institute, 2022). Worldwide, CAD is the second major cause of mortality, and its prevalence is equally high in South Asia. It has been estimated to affect up to 44% of the US adult population by the year 2030 (Ferreira-González, 2014; Dar et al., 2018). In Pakistan, CAD prevalence is about 11.2% in the local population (in females, it is 13.3%, and in males, 7.9%) (Dar et al., 2018). As compared to other ethnic groups, the people of South Asia are more prone to the development of atherosclerosis and thus have a high mortality rate (Nadeem et al., 2013). In the treatment of patients with CAD, major goals are to decrease the incidence of major adverse cardiac events (MACEs) that includes the composite of all-cause death, stent thrombosis (ST), myocardial infarction (MI), target lesion/vessel revascularization (TLR/TVR), and restenosis; improve symptoms, quality of life (QoL), and functional status; and to prolong life (Adnan et al., 2017; Zibaeenezhad et al., 2019).

If medical treatment for CAD is inappropriate or fails, there are two invasive procedures; one is the coronary artery bypass graft (CABG), the major cardiac surgery, and another is the balloon angioplasty or percutaneous transluminal coronary angioplasty (PTCA). PTCA involves the use of a balloon catheter for non-surgical widening of the artery. Recently, stents are being used in most of the PTCA procedures. Stents are composed of a thin wire-mesh platform which acts as a permanent prosthetic lining for keeping an artery inflated and maintaining its patency (excellence, 2003). The incidence of morbidity and mortality in patients with CAD has been reduced substantially by percutaneous coronary intervention (PCI). In the 1980s, bare metal stents (BMS) proved superior to balloon angioplasty with improved clinical outcomes and angiographic results. Later on, to decrease revascularization and neointimal hyperplasia associated with BMS, drug-eluting stents (DES) were designed in 2001 (Adnan et al., 2017; Dar et al., 2018; Lee and de la Torre Hernandez, 2018).

The coronary stent industry is growing on a rapid pace. There are many disadvantages of second-generation durable polymer drug-eluting stents (DP-DES) including the presence of a permanent polymer. The third-generation biodegradable polymer (BP) stents resolve the short-comings of DP-DES by leaving a polymer-free stent after completion of the anti-proliferative drug release process (Mehta et al., 2013; El-Hayek et al., 2017; Lee and de la Torre Hernandez, 2018; Sakamoto et al., 2018; Bangalore, 2019; Picard et al., 2019). Degradation completes in a duration of 3 to 15 months. Moreover, BP-DES are cost-effective as compared to the DP stents (Tsai et al., 2017). After PCI in patients with CAD, MACEs are the important reason of morbidity and mortality. To improve longevity and health, detection of the risk factors of MACE and their treatment is very crucial (Tsai et al., 2017). The major risk factors that have an impact on post-PCI outcomes in patients with CAD are smoking, hypertension (HTN), hyperlipidemia, and diabetes mellitus (DM) (Lin et al., 2017; Tsai et al., 2017; Wiemer et al., 2017; Kim et al., 2019).

Currently, the studies available on PCI have mainly focused on outcomes. Encouraging results have been found in the randomized controlled trials (RCTs), but longer duration follow-up studies awaited these newer generation stents (Nogic et al., 2018). Likewise in Pakistan, studies are available on the treatment intervention and assessment of therapeutic/adverse outcomes; however, there is a dearth of literature on the comparative studies of MACEs in post-PCI and post-primary-PCI (PPCI) patients. In the wake of multiplicity of options in the stent industry, decision makers need access to evidence-based information. Therefore, our study was designed to compare the MACEs in CAD patients undergoing PPCI with second- or third-generation DES and to evaluate the predictors of MACEs. This would be the first comparison-based study on BioMatrix and XIENCE stents in the Pakistani population.

## Methods

### Study design

An observational, cohort study was designed to assess MACEs in patients with CAD after PPCI with second-generation (Abbott’s Everolimus-Eluting XIENCE Prime/XIENCE Xpedition) or third-generation (Biosensors’ Biolimus-Eluting BioMatrix NeoFlex/BioMatrix Alpha) DES. The study consisted of two phases: a retrospective phase (in which data were retrieved from the hospital record) and a prospective phase (follow-up of patients at 1 year duration, post-PCI). The major adverse cardiovascular events are defined as the composite endpoints of non-fatal MI, stent thrombosis, clinically driven TVR/TLR, and cardiovascular death (Zibaeenezhad et al., 2019). The XIENCE Xpedition Everolimus-Eluting Stent (EES) having a cobalt chromium strut loaded with 100 µg/cm^2^ everolimus and XIENCE Prime (Abbott) is also a 100 µg/cm^2^ everolimus-coated stent (Abbott, 2013; Abbott, 2015). The BioMatrix Biolimus-Eluting Stent (BES) has a polylactic acid (PLA) biodegradable polymer (Biosensors International, Switzerland) (Separham et al., 2011). The BioMatrix NeoFlex is indicated in ST-elevated myocardial infarction (STEMI) patients, acute coronary syndrome (ACS), and diabetic patients. BioMatrix was first approved in 2015 (BIOSENSORS, 2020). The current study has been conducted at the Armed Forces Institute of Cardiology/National Institute of Heart Diseases (AFIC-NIHD), Rawalpindi, Pakistan. AFIC-NIHD is the country’s leading tertiary care cardiac center accredited with RCSEP for cardiac surgery training.

### Study population and subjects

All participants complying with the inclusion and exclusion criteria were recruited in the study. Inclusion criteria: All patients undergoing PPCI procedure at the study site between July and December 2019, patients with age 18 years or older, and those who have received either the second-generation XIENCE Prime/XIENCE Xpedition or the third-generation BioMatrix NeoFlex/BioMatrix Alpha stents were included in this study. There was no restriction regarding the type or length of the lesion. Exclusion criteria: All patients under 18 years of age, who have undergone PPCI for any other disease except CAD (arrhythmic diseases, cardiomyopathies, cardiac valvulopathies, *etc.*); received BMS, first-generation DES, or second-/third-generation stents except for XIENCE or BioMatrix; have previous history of PCI/PPCI, CABG, or plain old balloon angioplasty (POBA); and those who have received multiple stents were excluded.

**FIGURE 1:**
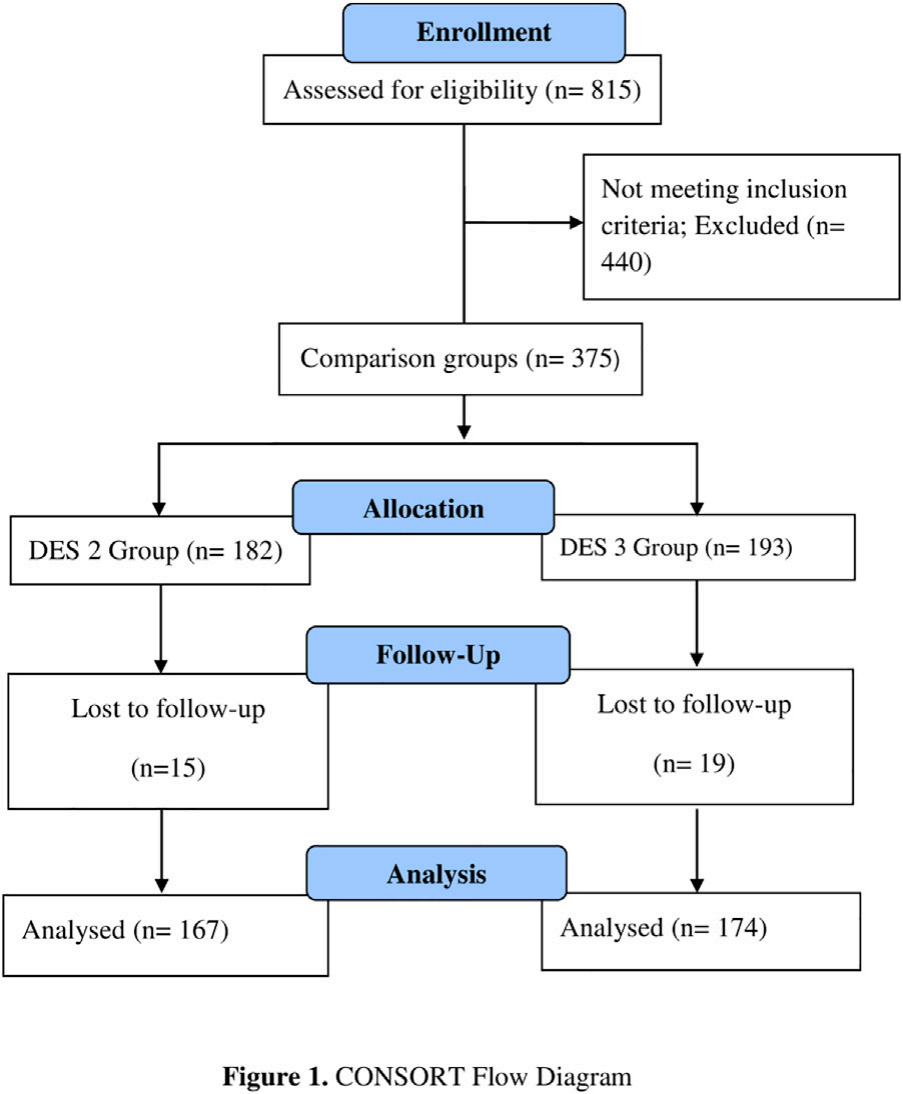
CONSORT flow diagram.

The sampling technique was non-probability convenience sampling. Sample size calculation was not performed, and all the participants meeting inclusion/exclusion criteria were recruited in the study [however, *a priori* power analysis was conducted, and keeping the power (1—β) value of 0.8, the calculated sample size was 169 patients in each group]. A total of 815 participants were screened according to the inclusion/exclusion criteria, who had undergone PPCI at the study site from July to December 2019. Those not fulfilling the inclusion criteria were dropped, and only 341 patients were enrolled. The participants were assigned to their respective groups, that is, second- or third-generation DES on the basis of the stent type they had received in the past on cardiologist discretion (167 in the second-generation XIENCE group and 174 in the third-generation BioMatrix group) (Figure 1).

### Operational definitions

Primary percutaneous coronary intervention refers to the strategy of taking STEMI patients directly to the cardiac catheterization laboratory to proceed mechanical revascularization using the balloon angioplasty, aspiration thrombectomy, coronary stents, and other measures (Levine, 2014). Myocardial infarction is the increase in myocardial necrosis biomarkers above the upper range limit associated with at least one of these conditions: development of the Q-wave on electrocardiography, ischemic symptoms, and the ECG changes that indicate ischemia (El-Hayek et al., 2017). Restenosis is the reduction in the diameter of lumen post-PCI, and it usually occurs between 3 and 12 months after stenting. Stent thrombosis has been defined as the presence of a thrombus that originates in the scaffold/stent or in the 5-mm distal or proximal segment to the scaffold/stent or in the side branch that originates from the scaffold/stented segment and the presence of one of these criteria; the new electrocardiograph (ECG) changes, suggesting acute ischemia, acute onset of the ischemic symptoms at rest, or typical rise or fall in the cardiac biomarkers. TLR is the repeated percutaneous intervention or the bypass surgery procedure of target vessels performed due to restenosis or any other complication of the targeted lesion. TVR is defined as the repeat surgical bypass or PCI of any portion of the target vessel or the target lesion (Garcia-Garcia et al., 2018). Cardiovascular death was defined as the death caused by any cardiac issue (e.g., heart failure, MI, or fatal arrhythmia) and unknown or un-witnessed death (Maupas et al., 2017).

### Data collection method

A data collection form was designed to obtain the patient’s history and demographic details. Angiographic characteristics, stent type, and reason for PPCI were retrieved from patients’ record at the hospital. At 1 year post-PPCI, all patients were interviewed during their follow-up visits to evaluate the past medical history and assessment of risk factors. Clinical outcomes were recorded by accessing MACEs in 1 year duration, that is, the incidence of MI, ST, TVR, TLR, and death. In-hospital MACEs (post-stenting procedure) were not assessed. and only after discharge, MACEs were included.

### Statistical analysis

Data evaluation was carried out by using the statistical software package for social sciences (IBM SPSS statistics version 21). Numbers and percentages were calculated for categorical variables, and the chi-squared test was applied for comparison. Continuous variables’ data were presented as the mean and standard deviation (SD) and the Mann–Whitney U test was used for the calculation of the *p*-value. MACE data were presented as frequencies and percentages. The association of MACEs with the demographic, angiographic, and risk factors was assessed by binary logistic regression analysis using the Wald test. Univariate analysis was performed first, and those variables having a *p*-value ˂0.25 were assessed again *via* multivariate analysis. The odds ratio and *p*-value were assessed, and the *p*-value less than 0.05 was considered statistically significant.

## Results

### Demographic and angiographic characteristics of the study population

Demographics of the study population (i.e., gender, age, residence, education, and occupation status) are presented as frequencies and percentages. The study population consisted of more male participants (86.2%) than female participants (13.8%); 52.2% patients were in the age group of 58–75 years (Table 1).

**TABLE 1:** Demographic characteristics of the study participants.

Demographic variable		Second-generation DES group (*n* = 167)	Third-generation DES group (*n* = 174)	Total (*n* = 341)	*p*-value *
Gender	Male	143 (85.6%)	151 (86.8%)	294 (86.2%)	0.641
	Female	24 (14.4%)	23 (13.2%)	47 (13.8%)	
Age (years)	19–38	3 (1.8%)	4 (2.3%)	7 (2.1%)	0.056
	39–57	83 (49.7%)	63 (36.2%)	146 (42.8%)	
	58–75	75 (44.9%)	103 (59.2%)	178 (52.2%)	
	>75	6 (3.6%)	4 (2.3%)	10 (2.9%)	
Residence	Urban	132 (79%)	107 (61.5%)	239 (70.1%)	*p* < 0.001
	Rural	35 (21%	67 (38.5%)	102 (29.9%)	
Education	Illiterate	—	1 (0.6%)	1 (0.3%)	*p* < 0.001
	Primary	11 (6.6%)	28 (16.1%)	39 (11.4%)	
	Secondary	72 (43.1%)	95 (54.6%)	167 (49%)	
	Intermediate	27 (16.2%)	28 (16.1%)	55 (16.1%)	
	Graduate	57 (34.1%)	22 (12.6%)	79 (23.2%)	
Occupation	Unemployed	82 (49.1%)	79 (45.4%)	161 (47.2%)	0.494
	Employed	85 (50.9%)	95 (54.6%)	180 (52.8%)	

*The chi-squared test is used for the calculation of the *p*-value.

Angiographic characteristics of the study participants include the type of MI, culprit artery (the treated vessel), CAD diagnosis, access site, and length of the stent. Summary statistics of these categorical variables is presented as frequencies and percentages except for stent length that is calculated as the mean value and standard deviation (Table 2). The type of MI on ECG was categorized as anterior MI (67.4%) and inferior MI (32.55%). Four categories were made based upon the treated vessel which are the left anterior descending coronary artery (LAD), right coronary artery (RCA), left circumflex coronary artery (LCX), and obtuse marginal branches (OM branch). In majority of the participants, the treated vessel was the LAD (55.1%). CAD types diagnosed in the study population were the single-vessel CAD (SVCAD) 38.4%, double-vessel CAD (DVCAD) 32.4%, and triple-vessel CAD (TVCAD) 29.1% (*n* = 99). The stent’s length range was 14–74 mm with the mean 28.86 mm (28.86 ± 8.679), for the second-generation DES, the mean value was 29.39 ± 9.825 and 28.86 ± 7.451 for the third-generation DES.

**TABLE 2:** Angiographic characteristics of the study participants.

Angiographic characteristic		Second-generation DES group (*n* = 167)	Third-generation DES group (*n* = 174)	Total (*n* = 341)	*p*-value*
Type of MI	ANT MI	108 (64.67%)	122 (70.11%)	230 (67.45%)	0.283
	INF MI	59 (35.33%)	52 (29.89%)	111 (32.55%)	
Culprit artery	LAD	87 (52.1%)	101 (58.04%)	188 (55.1%)	0.079
	RCA	54 (32.3%)	60 (34.4%)	114 (33.4%)	
	LCX	15 (8.9%)	10 (5.74%)	25 (7.3%)	
	OM branch	11 (6.59%)	3 (1.73%)	14 (4.1%)	
CAD diagnosis	SVCAD	62 (37.13%)	69 (39.66%)	131 (38.5%)	0.89
	DVCAD	54 (32.33%)	56 (32.2%)	110 (32.4%)	
	TVCAD	50 (29.9%)	49 (28.16%)	99 (29.1%)	
Access site	Radial	167	174	341 (100%)	**
Stent length (mm)	—	29.39 ± 9.825	28.36 ± 7.451	28.86 ± 8.679	0.274

ANT MI, anterior MI; INF MI, inferior MI; LAD, left anterior descending artery; LCX, left circumflex artery; RCA, right coronary artery; OM branch, obtuse marginal branch; SVCAD, single-vessel coronary artery disease; DVCAD, double-vessel coronary artery disease; TVCAD, triple-vessel coronary artery disease. * Chi-squared test is used for calculation of the *p*-value; for the continuous variable “stent length,” the Mann–Whitney *t*-test was used. **no statistics as the access site is constant.

### Risk factor assessment

The risk factors assessed for MACEs include a family history of CAD, smoking status, DM, HTN, and hyperlipidemia. Frequencies and percentages were calculated for these categorical variables. Summary statistics is presented in Table 3.

**TABLE 3:** Risk factors in the study participants.

Risk factor of MACEs		Second-generation DES group (*n* = 167)	Third-generation DES group (*n* = 174)	Total (*n* = 341)	*p*-value *
Family history of CAD	Yes	53 (31.7%)	46 (26.4%)	99 (29%)	0.401
	No	114 (68.3%)	128 (73.6%)	242 (71%)	
Ex-smoking history	Yes	34 (20.36%)	35 (20.11%)	69 (20.2%)	0.307
	No	133 (78.7%)	139 (79.89%)	272 (79.8%)	
Current smoking status	Yes	27 (16.2%)	36 (20.7%)	63 (18.5%)	0.282
	No	140 (83.8%)	138 (79.3%)	278 (81.5%)	
Diabetes mellitus	Yes	33 (19.8%)	39 (22.4%)	72 (21.1%)	0.548
	No	134 (80.2%)	135 (77.6%)	269 (78.9%)	
Hypertension	Yes	60 (35.92%)	53 (30.46%)	113 (33.1%)	0.284
	No	107 (64.07%)	121 (69.54%)	228 (66.9%)	
Hyperlipidemia	Yes	32 (19.2%)	38 (21.8%)	70 (20.5%)	0.629
	No	135 (8.8%)	136 (78.2%)	271 (79.5%)	

*Chi-squared test is used for the calculation of the *p*-value.

Summary statistics shows that about 29% people were having a previous history of CAD, more patients in the second-generation than in the third-generation DES group. About 19.1% people were ex-smokers, more in the third-generation DES group (*n* = 35), while 16.7% patients were also current smokers. DM^+^ patients were 21.1% (*n* = 72), more in the third-generation DES group (*n* = 39), while hypertensive patients were more in the second-generation DES group (*n* = 59); total HTN^+^ patients were 33.1%. Hyperlipidemia was found in 20.5% patients; the number was more in the third-generation DES group (*n* = 38).

### Major adverse cardiovascular events

MACEs were categorized as MI, restenosis, stent thrombosis, TLR/TVR, and cardiac death. Frequencies and percentages are presented as follows (Table 4).

**TABLE 4:** Major adverse cardiovascular event distribution in the second- and third-generation DES.

MACE	Second-generation DES group (*n* = 167)	Third-generation DES group (*n* = 174)	Total (*n* = 341)
MI	2 (1.19%)	1 (0.57%)	3 (0.9%)
ST	3 (1.80%)	2 (1.15%)	5 (1.5%)
Restenosis	2 (1.19%)	2 (1.15%)	4 (1.2%)
Cardiac death	1 (0.59%)	1 (0.57%)	2 (0.59%)
Non-cardiac death	2 (1.19%)	3 (1.72%)	5 (1.5%)
No MACE reported	157 (94.01%)	165 (94.83%)	322 (94.4%)

MI: myocardial infarction, ST: stent thrombosis, TLR: target lesion revascularization, LVR: target vessel revascularization; DES: drug-eluting stents.

MACEs include adverse events in patients monitored at the follow-up after 12 months from the date of PPCI (after discharge until 1 year), and in-hospital MACEs (during the hospital stay after the procedure) were not included. One MI case was reported in the third-generation DES group and two in the second-generation group. ST occurred in five patients (1.5%); a greater number of ST cases were observed in the second-generation DES (1.79%) than in the third-generation DES (1.15%) group, and no TVR/TLR was reported. The restenosis rate and cardiac death were almost the same in both groups: restenosis 1.19% vs. 1.15% and cardiac death 0.59% vs. 0.57% in the second- and third-generation DES groups, respectively. In 94.4% study population, no MACEs were reported at 1-year follow-up. Overall, the percentage of MACE was 4.19%; in the second-generation DES, it was 4.77%, and in the third-generation DES, it was 3.44%.

Binary logistic regression analysis (Wald test) was performed to find the association of MACEs with risk factors (i.e., DM, HTN, smoking, hyperlipidemia, and family history), demographic factors (age and gender), and angiographic variables (CAD diagnosis and the type of MI). Univariate analysis was performed first; those variables having a *p*-value ˂0.25 were assessed again *via* multivariate analysis (Table 5). The DES type, although not significant, was included in multivariate analysis due to its importance in the model. Overall, the logistic regression model was significant χ^2^ (8) = 38.211 and *p* < 0.0005. The model explained 36.6% variance (Nagelkerke R square) in the MACE and correctly classified 96.2% of cases.

**TABLE 5:** Factors associated with the occurrence of MACEs.

Sr No	Variable	Univariate analysis			Multivariate analysis		
		Odds ratio [exp (B)]	CI [95% CI for exp (B)]	*p*-value^a^	Odds ratio [exp (B)]	CI [95% CI for exp (B)]	*p*- value^a^
1	Age (Yes)	1.0	b*	0.99	—	—	—
	(No)	Ref			—		
2	Gender (Yes)	1.795	0.481–6.694	0.384	—	—	—
	(No)	Ref			—		
3	DM (Yes)	1.456	1.181–1.796	<0.001	0.226	0.061–0.828	0.025
	(No)	Ref.			Ref		
4	HTN (Yes)	1.125	0.925–1.371	0.005	0.212	0.050–0.894	0.035
	(No)	Ref.			Ref		
5	Current smokers (Yes)	1.291	0.893–1.692	0.023	0.141	0.033–0.603	0.008
	(No)	Ref			Ref		
6	Family history (Yes)	1.312	1.043–1.649	0.006	0.209	0.057–0.762	0.018
	(No)	Ref			Ref		
7	Hyperlipidemia (Yes)	1.007	0.816–1.243	0.161	0.474	0.165–2.307	0.474
	(No)	Ref			Ref		
8	DES type (BioMatrix)	1.409	0.478–4.15	0.534	0.297	0.142–1.813	0.297
	(XIENCE)	Ref			—		
9	CAD diagnosis (SVCAD)	5.474	0.602–49.758	0.131	0.069	0.008–1.203	0.069
	(DVCAD)	0.473	0.141–1.586	0.225	2.035	0.519–7.98	0.308
	(TVCAD)	Ref			Ref		
10	Type of MI (ANT MI)	1.158	0.379–3.541	0.797	—	—	—
	(INF MI)	Ref			—	—	—
11	Stent length	0.988	0.931–0.988	0.684	—	—	—

a: *p* < 0.05 is considered significant (binary logistic regression analysis has been performed to find the association of MACE with risk factors); b*: non-computable.

No high multi-collinearity was observed among predictors as assessed through the correlation matrix; all correlation coefficient values were below 0.90. A significant association (*p* < 0.05) was found between MACE, DM, HTN, current smoking, and family history. diabetes mellitus [*p* = 0.025, 95% CI: 0.061–0.828, Exp (B) = 0.226]; odds of having MACE in DM^+^ patients are 0.226 times more than non-DM patients. Hypertension [*p* = 0.035, 95% CI: 0.050–0.894, Exp (B) = 0.212]; odds of MACE in HTN^+^ patients were 0.212 times more than non-hypertensive patients. The current smoking status [*p* = 0.008, 95% CI: 0.033–0.603, Exp (B) = 0.141] and family history [*p* = 0.018, 95% CI: 0.057–0.762, Exp (B) = 0.209]. Although a greater number of MACE cases were reported in the second-generation DES (*n* = 8) than the third-generation DES group (*n* = 6), statistical analysis has not found any significant association (*p* = 0.297).

## Discussion

Newer generation BP-DES have been presented as a standard of care in PPCI. Various studies have compared the BP-DES with DP-DES; however, to the best of our knowledge, current study is the first comparative study of the third-generation BP-BioMatrix stents (BioMatrix Alpha and BioMatrix NeoFlex) and the second-generation DP-XIENCE stents (XIENCE Prime and XIENCE Xpedition) to evaluate the MACE in patients with CAD, post-PPCI, in the Pakistani population. The results of the current study found no significant difference in the rate of MACE between two stent types; however, the overall percentage of MACEs was more in XIENCE stents (4.75%) than in BioMatrix (3.44%) stents.

The number of male patients was much more than females in our sample (85.6%), and same findings were reported in several other PPCI studies [76.2% (Shah et al., 2018), 86% (Mian, 2014), and 81.8% (Mehta et al., 2013)]. The result of this study found that BP-BioMatrix stents are having similar or superior outcomes than DP-XIENCE stents at 1-year follow-up. These outcomes are in agreement with many other studies (Tsai et al., 2017; Park and Rha, 2020). Comparable results of BP-EES (XIENCE) and BP-sirolimus-eluting stents (SES) (orsiro) were obtained at 12 months follow-up in a RCT; TLR was same in both groups (*p* = 0.58) (Windecker et al., 2015). Safety and efficacy of the second-generation EES (XIENCE Prime, XIENCE V, and Promus), BES (BioMatrix, BioMatrix Flex, and Nobori), and zotarolimus-eluting stents (ZES) (resolute integrity/resolute) were compared in a study, and no significant association was found in the statistical analysis (Park et al., 2013). A prospective, randomized controlled, follow-up study found similar/comparable MACE of BP-biolimus-eluting stents (BioMatrix) and DP-EES (XIENCE-V) at 12 months follow-up (Separham et al., 2011).

Many other studies have also evaluated same outcomes; the NEXT trial compared BES (Nobori) with EES (XIENCE/Promus), follow-up after 1 year found the non-inferiority of BES (Natsuaki et al., 2013). A multicenter grand-DES registry compared efficacy and safety of BES (BioMatrix/BioMatrix Flex/Nobori), EES (XIENCE Prime/XIENCE V/Promus), and ZES (resolute integrity/resolute) and obtained comparable outcomes (Ki et al., 2020). Another study also concluded that there is no significant difference between MACEs of DP-EES and BP-BES (2.7% vs. 2.7%: *p* = 0.984) (Parsa et al., 2016). The COMPARE II trial found that the overall percentage of MACEs was more in BP than DP stents; however, results were not statistically significant (Vlachojannis et al., 2017). Comparison of long-term clinical outcomes of BP and DP stents by Tsai et al. (2020) has found no significant difference in MACEs (Smith et al., 2019). Women are reported to have a higher risk of developing adverse events; however, in our study, no significant association was found between MACEs and gender (Giustino et al., 2016).

Results of some studies showed the superiority of BP-DES to DP-DES (Mattke et al., 2019). A study reported that BP-SES were associated with the lower rate of MACEs than DP-DES at 1 year follow-up (Mian, 2014). Another study was carried out to evaluate the MACE of BP-EES and reported 3.8% MACE at follow-up. In India, a multicenter trial found 0.45% MACEs in BioMatrix BES at 1 year follow-up (Mehta et al., 2013; Shah et al., 2018). The LEADERS trial is a multicenter, 5 -year follow-up study comparing outcomes of BES with SES. Results showed that BES have superior safety and efficacy than SES (Zhang et al., 2015). In French e-BioMatrix registry, the MACE percentage was lower than the LEADERS trial of sirolimus-eluting cypher stents (DP-DES). The LEADERS randomized trial concluded that BP stents were having better safety and efficacy than DP-DES (Stefanini et al., 2012; Maupas et al., 2017). Toru et al. (2018) have compared vascular response of the second- and third-generation DES, in terms of quality and quantity, and concluded that the third-generation stents might have better long-term clinical outcomes (Miyoshi et al., 2018). Major adverse cardiovascular and cerebrovascular events (MACCEs) of second-generation XIENCE and third-generation synergy were compared in a RCT; results have found 19% of MACCEs in XIENCE and 16% in synergy (Walsh, 2018).

On the contrary, in some studies, results were not comparable to the findings of the current study. SORT OUT V: a randomized non-inferiority trial concluded that biodegradable polymer stents were not associated with better outcomes than DP stents (Christiansen et al., 2013). Results of a study comparing DP-EES and BP-SES showed that the rate of TLR was more in BP stents at a follow-up of 386 days (Kakizaki et al., 2020). The significant association of MACEs has been found with many factors like hypertension, smoking, hyperlipidemia, DM, and a family history of CAD. Our study evaluated the association of MACEs with various risk factors, that is, DM, smoking status, HTN, CAD family history, and hyperlipidemia. In statistical analysis, the significant association of MACEs was obtained with DM (*p*-value; 0.025), HTN (*p*-value; 0.035), family history of CAD (*p*-value; 0.018), and the current smoking status (*p*-value; 0.008). A higher risk of restenosis, mortality, and re-vascularization has been reported in diabetic patients (Seth et al., 2013).

A prospective study compared MACEs in diabetic and non-diabetic patients in Peshawar; the ratio of MACEs was more in the diabetic group, but a statistically significant association was not obtained (Adil et al., 2021). In another study in India, MACEs associated with BES in diabetic patients were evaluated (Seth et al., 2013). Although e-BioMatrix French registry evaluated MACEs in biolimus stents, the rate of MACE was same in diabetic and non-diabetic populations (Maupas et al., 2017). BP-SES and DP-EES were compared in diabetic patients, and results concluded that BP stents were associated with more TLR than DP stents in diabetic people (Kakizaki et al., 2020). The percentage of MACE was more in DM and hypertensive patients at a follow-up of 66.5 months, and the combined effect of DM and HTN increased the incidence of MACEs further (Zibaeenezhad et al., 2019).

Another study evaluated the long-term impact of DM and HTN. Results found that mortality and MI were highest in the DM group (*p* < 0.001) as compared to HTN and HTN + DM groups (Lin et al., 2017). Evidence of smoking association with the increased rate of MACEs after implantation of DES was also found in a study carried out at Peshawar Hospital (Adnan et al., 2017). A study to evaluate the smoking impact on MACEs was conducted in Korea that concluded similar efficacy and safety in smokers vs. non-smokers (Kim et al., 2018). MACE and smoking association were accessed in a meta-analysis, which found that smoking is not associated with MACE (Hu et al., 2015). This is contradictory to the current study results, where a significant association has been found between smoking and MACEs. A retrospective study in Tehran was conducted to recognize major predictors of MACE after PCI and found that DM (*p* = 0.007) and CAD family history (*p* = 0.003) were risk factors of MACEs. The study was conducted on elderly patients (age ≥65 years) (Aghajani et al., 2018).

### Study limitations

This study has been conducted at a single center, so the sample may not be the representative of the whole CAD population. Stent selection bias may exist due to retrospective nature. Severity of disease was also not accounted in the study.

## Conclusion

Newer generation BP-DES have been introduced as a novel solution to the problems of durable polymer stents. Our study has compared the safety and efficacy of BP-BioMatrix stents with the older DP-XIENCE DES and evaluated the major predictors of MACEs. Biodegradable polymer stents were found to have comparable or superior efficacy and safety than the durable polymer stents at 1 year follow-up duration. Results demonstrated non-inferiority of BP-DES. However, studies with a longer follow-up, larger sample size, and randomized trials are required to better define comparative MACEs in both groups. Significant predictors of MACEs were hypertension, diabetes mellitus, smoking, and family CAD history.

### Future Perspective

Results of the current study will assist the policy makers and healthcare providers in the rationalization of scarce resources and will provide information about the new biodegradable polymer stents. However, RCTs with longer follow-up duration are required for convincing evidence.

## Data Availability

The original contributions presented in the study are included in the article/supplementary material, further inquiries can be directed to the corresponding authors.

## Abbreviations

BES, biolimus-eluting stent; BP, biodegradable polymer; CAD, coronary artery disease; DES, drug-eluting stents; DP, durable polymer; EES, everolimus-eluting stent; HTN, hypertension; MACE, major adverse cardiovascular event; MI, myocardial infarction; PPCI, primary percutaneous coronary intervention; PTCA, percutaneous transluminal coronary angioplasty; ST, stent thrombosis; STEMI, ST-elevated myocardial infarction; TLR, target lesion revascularization; TVR, target vessel revascularization.
